# Estimation for the *P*(*X* > *Y*) of Lomax distribution under accelerated life tests

**DOI:** 10.1016/j.heliyon.2024.e25802

**Published:** 2024-02-07

**Authors:** Hassan M. Aljohani

**Affiliations:** Department of Mathematics and Statistics, College of Science, Taif University, P.O. Box 11099, Taif 21944, Saudi Arabia

**Keywords:** Stress-strength reliability, Lomax distribution, Classical estimation, Bayesian estimation

## Abstract

The system or unit survives when strength is more significant than the stress enjoined. This procedure is usually used in many companies to test their product. The reliability or the quality of the scheme or component is described by the parameters of stress-strength reliability (R=P(X>Y)) where *X* denotes strength and *Y* indicates stress. In this article, we adopted the statistical inference of *R* while the two arbitrary factors *X* and *Y* are independent and approach the Lomax lifetime distribution with common scale parameters. Also, the strength and stress variables are subjected to a partial step-stress-quickened life experiment. The classical estimation and Bayes method create the point estimate of *R*. Confidence intervals of *R* are computed with asymptotic distribution, bootstrap technique, and Bayesian credible intervals. All results are evaluated and compared under an extensive simulation study. Finally, the lifetime data sets generated from the Lomax distribution are used to analyze the system's reliability by estimating *R*.

## 1 Introduction

The Lomax distribution (LD) has two parameters, *β*, and *α*, and it is a heavy-tailed probability density distribution proposed early by Lomax [1]. The LD is a popular distribution to several applications such as internet traffic modeling, actuarial science, business, economics, and queueing theory, see Marshall and Olkin [2]. Also, LD is known as the Pareto Type-II distribution and is utilized in several situations of lifetime analysis, Chahkandi and Ganjali [3]. LD with the property of heavy and decreasing failure rate function was utilized as an alternative to indexing exponential, Weibull, and gamma distributions. For more details, see Afify et al. [4], Bryson [5], Teamah et al. [6], Alfaer et al. [7] and Raqab et al. [8].

The random variable *T* has a Lomax distribution with cumulative distribution function (CDF) is defined as

(1) $$F(t)=1-{\left(1+\frac{t}{\beta }\right)}^{-\alpha },\;t>0,\;\alpha ,\beta >0,$$

where *α* and *β* are shape and scale parameters. LD was used for modeling lifetime data; for more detail, Cramer and Schmiedt [9] discussed the statistical inference of LD under competing risks scheme. Also, Tahani et al. [10] adopted the statistical inference of LD under partially observed causes of failure and Alghamdia et al. [11] have discussed the statistical inference of accelerated LD with copula approach. For more studies on LD, see [12], [13].

The scheme only works in literature when the applied strength is less than the stress. Therefore, the issue of estimating *R* appears as a measure of the mechanical reliability of a scheme or units. Estimation of *R*, when the lifetimes of two machines are presented by two variables *X* and *Y*, estimates the probability that one fails before the other. To clarify further, suppose we have two classes of electrical cable insulation put under failure voltage levels in a laboratory test. The two types are subjected to increasing voltage stress. Determining the class of insulation that has a longer life is our objective. Also, the lifetimes of type 1 and 2 insulation are expressed by variables *X* and *Y*, respectively. If, the value of R=P(X>Y) greater than 0.5 means that Type 1 insulation is superior in longevity. The issue of assessment of *R* was concerned early in nonparametric and parametric forms by different authors. For example, AL-Hussaine et al. [14] presented this problem under finite mixtures of lognormal components, Surles and Padgett [15] discussed the inference for *R* from Burr-Type *X* distribution, Nadarajah [16] discussed *R* under Laplace distribution, Kundu and Gupta evaluated *R* for the generalised exponential distribution– see [17] for more details, Hussam et al. [18] discussed fuzzy and traditional stress-strength reliability for inverse Weibull distribution, Mokhlis debated *R* model from Burr Type-III distributions [19], Alsadat et al. received Bayesian and non-Bayesian strategies with the MCMC techniques of the stress-strength principle by using two new parameters of Poisson Rayleigh distribution–see [20] for more information. Kundu and Gupta [21] estimated *R* form Weibull distributions, Yousef et al. discussed strength element partially accelerated to analyze stress—strength principle with simulation study– see [22] for more details, Krishnamoorthy and Mukherjee [23] discussed inference of *R* from two-parameter exponential distribution, Almetwally et al. [24] concerned a choice strategy of multi-stress–strength reliability using two approaches, Bayesian and non-Bayesian techniques, for the alpha power exponential principle employing progressive first failure, Salem et al. [25] discussed Bayesian and non-Bayesian approaches to evaluate reliability in general Type-II progressive hybrid analysis systems, Kundu and Raqab [26] estimated *R* from three-parameter Weibull distribution, Krishnamoorthy and Lin [27] discussed confidence limits for *R* from Weibull distribution, Mahmoud et al. [29] discussed *R* model from Weibull gamma distribution. Abd-Elmougod and Hanaa [30] estimated *R* under records for two-parameter bathtub shape distribution, Kumar and Siju [28] discussed *R* under exponential and Weibull. Recently, Sarhan and Tolba [31] discussed the accelerated strength of the *R* model from Weibull distribution.

Collecting information about the life of units is more difficult with highly reliable products. Therefore, the test under ordinary conditions carries an extended period. One of the most essential solutions is accelerated life tests (ALTs) to overcome this issue–see Nelson [32]. Other types of ALT exist, one known as step-stress ALT. In step-stress ALT, the applied stress changes over a fixed time or number. If the test first passes through normal stress conditions, we mean partially step-stress ALT. For more details about step-stress ALT, see Soliman et al. [33], Hussam et al. [34], El-Sherpieny et al. [35], Bantan et al. [36], Al-Essa et al. [37], and Abd El-Raheem et al. [38].

In the problem at hand, we consider the Lomax lifetime population and discuss the estimation problem of system reliability. Under consideration, two independent variables, *X* and *Y*, have Lomax lifetime populations with popular scale parameters and different shape parameters. The variable *X* denote to strength and *Y* denote to stress. The two data are collected under a partially step-stress ALT model. Therefore, we study the estimation of *R* with classical and Bayes methods. The proposed model is assessed under a formulated Monte Carlo study. Also, determine the reliability of real populations for a given real data set.

The article is categorised as follows. A stress-strength reliability pinnacle under a partially step-stress ALT model is formulated in Section 2. The estimate of *R* and asymptotic confidence interval by maximum likelihood method in Section 3. Parametric bootstrap-*p* and bootstrap-*t* confidence interval of *R* in Section 4. Bayesian approach to estimate *R* in Section 5. A different estimation method is discussed through extensive simulation study in Section 6. The suggested model is analysed operating real-life data in Section 6. Finally, the conclusion and statements are written in Section 7.

## 2 P(X>Y) with partially step-stress ALT model

Suppose that, two samples of sizes *m* and *n* random selected from the common scale Lomax distribution with PDFs $f_{x}(.)$ and $f_{y}(.)$, respectively are defined as

(2) $$f_{x}(x)=\frac{\alpha_{1}}{\beta }{\left(1+\frac{x}{\beta }\right)}^{-(\alpha_{1}+1)},\;x>0,\;\alpha_{1},\;\beta >0,$$

and

(3) $$f_{y}(y)=\frac{\alpha_{2}}{\beta }{\left(1+\frac{y}{\beta }\right)}^{-(\alpha_{2}+1)},\;y>0,\;\alpha_{2},\;\beta >0.$$

Also, suppose the two samples are placed simultaneously on partially step-stress ALT as shown in Eqs. (4) and (5). The samples were put under normal conditions until reaching stress-change time *η* then put under stress condition until the remaining units are failures. The collected information about the failure units describe as

(4) $$X=(X_{1},\;X_{1},\ldots ,X_{J_{1}},X_{J_{1}+1},\ldots ,X_{m}),\;X_{J_{1}}<\eta <X_{J_{1}+1},\;0\leq J_{1}\leq m,$$

and

(5) $$Y=(Y_{1},\;Y_{1},\ldots ,Y_{J_{2}},\;Y_{J_{2}+1},\ldots ,Y_{n}),Y_{J_{1}}<\eta <Y_{J_{1}+1},0\leq J_{2}\leq n.$$

The total test time *Z* under partially step-stress ALT as given by DeGroot and Goel [39] defined as

(6) $$Z=\left\{ \begin{array}{c} T,\;T\leq \eta \\ \eta +\frac{\left(T-\eta \right)}{\theta },\;T>\eta \end{array},\right.$$

and the corresponding probability density function is defined as

(7) $$f(z)=\left\{ \begin{array}{c} f_{1}(z),\;T\leq \eta \\ f_{2}(z),\;T>\eta \end{array},\right.$$

where *θ* is accelerated parameter and *η* is the stress change time.

### 2.1 The model considerations

Suppose that the strength variable *X* has a Lomax distribution with parameters $\alpha_{1}$ and *β* and stress variable *Y* has Lomax distribution with parameters $\alpha_{2}$ and *β*, say *X* ∼LD( $\alpha_{1}$, *β*) and stress variable *Y* ∼LD( $\alpha_{2}$, *β*). Under the partially step-stress ALT model, the function (2) and (3) reduced

(8) $$f_{x}(x)=\left\{ \begin{array}{c} f_{1x}(x)=\frac{\alpha_{1}}{\beta }{\left(1+\frac{x}{\beta }\right)}^{-(\alpha_{1}+1)},\;x\leq \eta \\ f_{2x}(x)=\frac{\alpha_{1}\theta }{\beta }{\left(1+\frac{\eta +\theta (x-\eta )}{\beta }\right)}^{-(\alpha_{1}+1)},\;x>\eta \end{array},\right.$$

and

(9) $$f_{y}(y)=\left\{ \begin{array}{c} f_{1y}(y)=\frac{\alpha_{2}}{\beta }{\left(1+\frac{y}{\beta }\right)}^{-(\alpha_{2}+1)},\;y\leq \eta \\ f_{2y}(y)=\frac{\alpha_{2}\theta }{\beta }{\left(1+\frac{\eta +\theta (y-\eta )}{\beta }\right)}^{-(\alpha_{2}+1)},\;y>\eta \end{array}.\right.$$

And the corresponding CDF is defined as

(10) $$F_{x}(x)=\left\{ \begin{array}{c} F_{1x}(x)=1-{\left(1+\frac{x}{\beta }\right)}^{-\alpha_{1}},\;x\leq \eta \\ F_{2x}(x)=1-{\left(1+\frac{\eta +\theta (x-\eta )}{\beta }\right)}^{-\alpha_{1}},\;x>\eta \end{array},\right.$$

and

(11) $$F_{y}(y)=\left\{ \begin{array}{c} F_{1y}(y)=1-{\left(1+\frac{y}{\beta }\right)}^{-\alpha_{2}},\;y\leq \eta \\ F_{2y}(y)=1-{\left(1+\frac{\eta +\theta (y-\eta )}{\beta }\right)}^{-\alpha_{2}},\;y>\eta \end{array}.\right.$$

### 2.2 Stress-strength model

The stress-strength reliability approach is the correct spelling of the word, of Lomax distribution according to the last model consideration is given by

(12) $$R=P(X>Y)=\int_{0}^{\eta }f_{1x}(x)F_{1y}(x)dx+\int_{\eta }^{\infty }f_{2x}(x)F_{2y}(x)dx.$$

Eq. (12) by using Eq. (8) to (11) is reduced to

(13) $$R=\int_{0}^{\eta }\frac{\alpha_{1}}{\beta }{\left(1+\frac{x}{\beta }\right)}^{-(\alpha_{1}+1)}\left[1-{\left(1+\frac{x}{\beta }\right)}^{-\alpha_{2}}\right]dx+\int_{\eta }^{\infty }\frac{\alpha_{1}\theta }{\beta }{\left(1+\frac{\eta +\theta (x-\eta )}{\beta }\right)}^{-(\alpha_{1}+1)}\left[1-{\left(1+\frac{\eta +\theta (x-\eta )}{\beta }\right)}^{-\alpha_{2}}\right]dx.=\frac{\alpha_{2}}{\alpha_{1}+\alpha_{2}}.$$

## 3 Maximum likelihood estimation of *R*

The joint likelihood distribution of two samples $X=\left(X_{1},\;X_{1},\ldots ,X_{J_{1}},\;X_{J_{1}+1},\ldots ,X_{m}\right)$ and $Y=(Y_{1},Y_{1},\ldots ,Y_{J_{2}},Y_{J_{2}+1},\ldots ,Y_{n})$, without normalized constant is given by

(14) $$f_{x,y}(x,y|\Theta )=\prod_{i=1}^{J_{1}}f_{1x}(x_{i})\prod_{i=J_{1}+1}^{m}f_{2x}(x_{i})\prod_{i=1}^{J_{2}}f_{1y}(y_{i})\prod_{i=J_{2}+1}^{n}f_{2y}(y_{i}),$$

where the parameter vector $\Theta =\{\alpha_{1}$, $\alpha_{2}$, *β*, *θ*}. The joint likelihood function (14) reduced to

$$L_{x,y}(\Theta |x,y)=\prod_{i=1}^{J_{1}}\frac{\alpha_{1}}{\beta }{\left(1+\frac{x_{i}}{\beta }\right)}^{-(\alpha_{1}+1)}\prod_{i=J_{1}+1}^{m}\frac{\alpha_{1}\theta }{\beta }{\left(1+\frac{\eta +\theta (x_{i}-\eta )}{\beta }\right)}^{-(\alpha_{1}+1)}\times \prod_{i=1}^{J_{2}}\frac{\alpha_{2}}{\beta }{\left(1+\frac{y_{i}}{\beta }\right)}^{-(\alpha_{2}+1)}\prod_{i=J_{2}+1}^{n}\frac{\alpha_{2}\theta }{\beta }{\left(1+\frac{\eta +\theta (y_{i}-\eta )}{\beta }\right)}^{-(\alpha_{2}+1)},$$

(15) $$L_{x,y}(\Theta |x,y)=\alpha_{1}^{m}\alpha_{2}^{n}\beta^{-(m+n)}\theta^{m+n-(J_{1}+J_{2})}\exp[-(\alpha_{1}+1)\{\sum_{i=1}^{J_{1}}\log\left[1+\frac{x_{i}}{\beta }\right]+\sum_{i=J_{1}+1}^{m}\log\left[1+\frac{\eta +\theta (x_{i}-\eta )}{\beta }\right]\}-(\alpha_{2}+1)\{\sum_{i=1}^{J_{2}}\log\left[1+\frac{y_{i}}{\beta }\right]+\sum_{i=J_{2}+1}^{m}\log\left[1+\frac{\eta +\theta (y_{i}-\eta )}{\beta }\right]\}].$$

The natural logarithms of (15) are become

(16) $$\ell_{x,y}(\Theta |x,y)=m\log\alpha_{1}+n\log\alpha_{2}-(m+n)\log\beta +\left(m+n-(J_{1}+J_{2})\right)\log\theta -(\alpha_{1}+1)\left\{\sum_{i=1}^{J_{1}}\log\left[1+\frac{x_{i}}{\beta }\right]+\sum_{i=J_{1}+1}^{m}\log\left[1+\frac{\eta +\theta (x_{i}-\eta )}{\beta }\right]\right\}-(\alpha_{2}+1)\left\{\sum_{i=1}^{J_{2}}\log\left[1+\frac{y_{i}}{\beta }\right]+\sum_{i=J_{2}+1}^{n}\log\left[1+\frac{\eta +\theta (y_{i}-\eta )}{\beta }\right]\right\}.$$

### 3.1 Point ML estimate of R

Derive Eq. (16) respected to Θ

(17) $${\overset{ˆ}{\alpha }}_{1}(\beta ,\theta )=\frac{m}{\sum_{i=1}^{J_{1}}\log\left[1+\frac{x_{i}}{\beta }\right]+\sum_{i=J_{1}+1}^{m}\log\left[1+\frac{\eta +\theta (x_{i}-\eta )}{\beta }\right]},$$

(18) $${\overset{ˆ}{\alpha }}_{2}(\beta ,\theta )=\frac{n}{\sum_{i=1}^{J_{2}}\log\left[1+\frac{y_{i}}{\beta }\right]+\sum_{i=J_{2}+1}^{n}\log\left[1+\frac{\eta +\theta (y_{i}-\eta )}{\beta }\right]},$$

(19) $$-(m+n)\beta +(\alpha_{1}+1)\left\{\sum_{i=1}^{J_{1}}\frac{x_{i}}{\left(1+\frac{x_{i}}{\beta }\right)}+\sum_{i=J_{1}+1}^{m}\frac{\eta +\theta (x_{i}-\eta )}{\left(1+\frac{\eta +\theta (x_{i}-\eta )}{\beta }\right)}\right\}+(\alpha_{2}+1)\left\{\sum_{i=1}^{J_{2}}\frac{y_{i}}{\left(1+\frac{y_{i}}{\beta }\right)}+\sum_{i=J_{2}+1}^{n}\frac{\eta +\theta (y_{i}-\eta )}{\left(1+\frac{\eta +\theta (y_{i}-\eta )}{\beta }\right)}\right\}=0$$

(20) $$\frac{\left(m+n-(J_{1}+J_{2})\right)\beta }{\theta }-(\alpha_{1}+1)\left\{\sum_{i=J_{1}+1}^{m}\frac{\left(x_{i}-\eta \right)}{1+\frac{\eta +\theta (x_{i}-\eta )}{\beta }}\right\}-(\alpha_{2}+1)\left\{\sum_{i=J_{2}+1}^{n}\frac{\left(y_{i}-\eta \right)}{1+\frac{\eta +\theta (y_{i}-\eta )}{\beta }}\right\}=0.$$

Eqs. (19) to (20) have shown that The ML equations are reduced to two non-linear equations translated by Newton–Raphson iteration to receive the estimate of $\overset{ˆ}{\beta }$ and $\overset{ˆ}{\theta }$. The ML estimate of the parameters $\alpha_{1}$ and $\alpha_{2}$ are received by exchange $\overset{ˆ}{\beta }$ and $\overset{ˆ}{\theta }$ in (17) and (18). The ML estimation of reliability *R* is written as

(21) $$\overset{ˆ}{R}=\frac{{\overset{ˆ}{\alpha }}_{2}}{{\overset{ˆ}{\alpha }}_{1}+{\overset{ˆ}{\alpha }}_{2}}.$$

### 3.2 Confidence interval estimate of *R*

From the log-likelihood function (16), the second derivative of the log-likelihood equation with respected to $\alpha_{1}$ and $\alpha_{2}$ is computed as

(22) $$I_{\alpha_{1}\alpha_{1}}=\frac{-m}{\alpha_{1}^{2}},$$

(23) $$I_{\alpha_{2}\alpha_{2}}=\frac{-n}{\alpha_{2}^{2}},$$

(24) $$I_{\alpha_{1}\alpha_{2}}=I_{\alpha_{2}\alpha_{1}}=0.$$

If, we consider the vector $\Phi =(\alpha_{1},\alpha_{2})$ Fisher information matrix of $\Phi =(\alpha_{1},\alpha_{2})$ is denoted by Ω($\alpha_{1},\alpha_{2}$),

(25) $$\Omega (\alpha_{1},\alpha_{2})=\left(\begin{array}{cc} \frac{m}{\alpha_{1}^{2}}\; & 0\\ 0\; & \frac{n}{\alpha_{2}^{2}} \end{array}\right),$$

the related inverse of the Fisher information matrix is delivered by

(26) $$\Omega^{-1}(\alpha_{1},\alpha_{2})=\left(\begin{array}{cc} \frac{\alpha_{1}^{2}}{m}\; & 0\\ 0\; & \frac{\alpha_{2}^{2}}{n} \end{array}\right).$$

Also, the delta strategy is used to receive the variance of *R*

(27) $$V_{R}={\left(\frac{\partial R}{\alpha_{1}}\right)}^{2}\Omega_{11}^{-1}+{\left(\frac{\partial R}{\alpha_{2}}\right)}^{2}\Omega_{22}^{-1}.$$

Therefore, Eq. (27) can be written as

(28) $$V_{R}=\left(\frac{m+n}{mn}\right){\left(\frac{\alpha_{1}\alpha_{2}}{{\left(\alpha_{1}+\alpha_{2}\right)}^{2}}\right)}^{2}.$$

Hence, (1-*γ*)100% confidence interval of *R* is delivered by

(29) $$\left(\overset{ˆ}{R}-Z_{\frac{\gamma }{2}}\sqrt{V_{R}},\;\overset{ˆ}{R}+Z_{\frac{\gamma }{2}}\sqrt{V_{R}}\right),$$

where $Z_{\frac{\gamma }{2}}$ is percentile normal variate *N*(0, 1) and the confidence level is given by *γ*.

## 4 Bootstrap confidence interval of *R*

In literature, bootstrap techniques are commonly used in estimating the parameters and calculating the bias or variance of estimators. The parametric and nonparametric techniques can be applied in the bootstrap approach; see Davison and Hinkley [40] and Efron [41]. The bootstrap method under a small sample size has presented more accurate confidence intervals, see DiCiccio and Efron [42]. In this section, we adopted bootstrap-*p* and bootstrap-*t* techniques as a parametric bootstrap method to formulate the confidence interval of *R* as the following algorithms.

### 4.1 Algorithm 1 (bootstrap-p confidence interval)

1 For given, *m*, *n*, *η* and the original two samples $X=\left(X_{1},\;X_{2},\ldots ,X_{J_{1}},X_{J_{1}+1},\ldots ,X_{m}\right)$ and $Y=(Y_{1}$, $Y_{2},\ldots ,Y_{J_{2}}$, $Y_{J_{2}+1},\ldots$, $Y_{n})$, compute two integers $J_{1}$, $J_{2}$ and the estimate $\overset{ˆ}{\Theta }=\{{\overset{ˆ}{\alpha }}_{1}$, ${\overset{ˆ}{\alpha }}_{2}$, $\overset{ˆ}{\beta }$, $\overset{ˆ}{\theta }\}$.
2 From the distributions (10) and (11) generate two bootstrap random variable $X^{⁎}=(X_{1}^{⁎}$, $X_{2}^{⁎},\ldots ,X_{J_{1}}^{⁎}$, $X_{J_{1}+1}^{⁎},\ldots ,X_{m}^{⁎}$) and $Y^{⁎}=(Y_{1}^{⁎}$, $Y_{2}^{⁎},\ldots ,Y_{J_{2}}^{⁎}$, $Y_{J_{2}+1}^{⁎},\ldots ,Y_{n}^{⁎})$.
3 Compute $J_{1}$ and $J_{2}$.
4 For the same values of *m*, *n* and *η* calculate the bootstrap methods ${\overset{ˆ}{\Theta }}^{⁎}=\{{\overset{ˆ}{\alpha }}_{1}^{⁎}$, ${\overset{ˆ}{\alpha }}_{2}^{⁎}$, ${\overset{ˆ}{\beta }}^{⁎}$, ${\overset{ˆ}{\theta }}^{⁎}\}$.
5 Calculate the bootstrap computation of *R*, say $R^{⁎}=\frac{{\overset{ˆ}{\alpha }}_{2}^{⁎}}{{\overset{ˆ}{\alpha }}_{1}^{⁎}+{\overset{ˆ}{\alpha }}_{2}^{⁎}}$.
6 The steps from (2) to (5) are repeated **M** times to obtain ${\overset{ˆ}{R}}^{⁎}=$($R_{1}^{⁎}$, $R_{2}^{⁎},\ldots ,R_{M}^{⁎}$).
7 Put the bootstrap sample estimate ${\overset{ˆ}{R}}^{⁎}=$($R_{1}^{⁎}$, $R_{2}^{⁎},\ldots ,R_{M}^{⁎}$) in ascending order as ${\overset{ˆ}{R}}^{⁎}=$($R_{\left(1\right)}^{⁎}$, $R_{\left(2\right)}^{⁎},\ldots ,R_{(M)}^{⁎}$).
8 Suppose, $\Psi (z)=P({\overset{ˆ}{R}}^{⁎}<z)$ is the empirical CDF of ordered ${\overset{ˆ}{R}}^{⁎}$ then ${\overset{ˆ}{R}}_{\text{boot-p}}^{⁎}(z)=\Psi^{-1}(z)$.
9 The (1-*γ*)100% percentile bootstrap confidence intervals of *R* defined as
  (30) $$\left({\overset{ˆ}{R}}_{\text{boot-p}}^{⁎}(\frac{\gamma }{2}),\;{\overset{ˆ}{R}}_{\text{boot-p}}^{⁎}(1-\frac{\gamma }{2})\right).$$

### 4.2 Algorithm 2 (bootstrap-*t* confidence interval)

1. From the order sample ${\overset{ˆ}{R}}^{⁎}=$($R_{\left(1\right)}^{⁎}$, $R_{\left(2\right)}^{⁎},\ldots ,R_{(M)}^{⁎}$) compute the order statistics as ${\overset{ˆ}{\delta }}^{⁎}=$($\delta_{\left(1\right)}^{⁎}$, $\delta_{\left(2\right)}^{⁎},\ldots ,\delta_{(M)}^{⁎}$)
  (31) $${\overset{ˆ}{\delta }}_{\left(i\right)}^{⁎}=\frac{R_{\left(i\right)}^{⁎}-\overset{ˆ}{R}}{\sqrt{V_{R}^{⁎}}},\;i=1,2,\ldots ,M,$$
  where $V_{R}^{⁎}$ given by (28).
2. Suppose, $\Psi (z)=P({\overset{ˆ}{\delta }}^{⁎}<z)$ is the empirical CDF of ordered ${\overset{ˆ}{Z}}^{⁎}$ then ${\overset{ˆ}{Z}}_{\text{boot-t}}^{⁎}(z)=\Psi^{-1}(z)$.
3. Compute the value ${\overset{ˆ}{R}}_{\text{boot-t}}^{⁎}=\overset{ˆ}{R}+\Psi^{-1}(z)\sqrt{V_{R}^{⁎}}$.
4. The (1-*γ*)100% bootstrap-t confidence intervals of *R* define by
  (32) $$\left({\overset{ˆ}{R}}_{\text{boot-t}}^{⁎}(\frac{\gamma }{2}),\;{\overset{ˆ}{R}}_{\text{boot-t}}^{⁎}(1-\frac{\gamma }{2})\right).$$

## 5 The Bayesian estimation using MCMC

In the Bayesian approach, we benefit from prior information in the form of prior distribution and the details in data in the form of a likelihood distribution. In this section, suppose that independent gamma prior for the parameters $\Theta =\{\alpha_{1}$, $\alpha_{2}$, *β*} and non-informative prior for accelerated factors as follows

(33) $$\Theta_{i}\to \text{Gamma}(a_{i},\;b_{i}),i=1,2,3,$$

and

(34) $$P^{⁎}(\theta )\propto \frac{1}{\theta }.$$

Generally, the posterior distribution is given by

(35) $$P^{⁎}(\Theta )=\frac{P^{⁎}(\Theta )\times L_{x,y}(\Theta |x,y)}{{⨌}_{\Theta }P^{⁎}(\Theta )\times L_{x,y}(\Theta |x,y)d\alpha_{1}d\alpha_{2}d\beta d\theta }.$$

The last posterior distribution needs to compute a high dimensional integral which can not obtained in theoretical form. Therefore, we adopt the proportional form obtained from (15), (34), and (35), as the posterior can be written as

(36) $$P(\Theta |x,y)\propto \alpha_{1}^{m+a_{1}-1}\alpha_{2}^{n+a_{1}-1}\beta^{-(m+n)+a_{3}-1}\theta^{m+n-(J_{1}+J_{2})-1}\exp\left[-b_{1}\alpha_{1}-b_{2}\alpha_{2}-b_{3}\beta \right]\times \exp\left[-(\alpha_{1}+1)\left\{\sum_{i=1}^{J_{1}}\log\left[1+\frac{x_{i}}{\beta }\right]+\sum_{i=J_{1}+1}^{m}\log\left[1+\frac{\eta +\theta (x_{i}-\eta )}{\beta }\right]\right\}\right]\times \exp\left[-(\alpha_{2}+1)\left\{\sum_{i=1}^{J_{2}}\log\left[1+\frac{y_{i}}{\beta }\right]+\sum_{i=J_{1}+1}^{m}\log\left[1+\frac{\eta +\theta (y_{i}-\eta )}{\beta }\right]\right\}\right].$$

The posterior distribution reduced to the full conditional distributions defined as

(37) $$P_{1}(\alpha_{1}|x,y)\propto \alpha_{1}^{m+a_{1}-1}\exp\left[-\alpha_{1}\left\{b_{1}+\sum_{i=1}^{J_{1}}\log\left[1+\frac{x_{i}}{\beta }\right]+\sum_{i=J_{1}+1}^{m}\log\left[1+\frac{\eta +\theta (x_{i}-\eta )}{\beta }\right]\right\}\right],$$

(38) $$P_{2}(\alpha_{2}|x,y)\propto \alpha_{2}^{n+a_{2}-1}\exp\left[-\alpha_{2}\left\{b_{2}+\sum_{i=1}^{J_{2}}\log\left[1+\frac{y_{i}}{\beta }\right]+\sum_{i=J_{2}+1}^{n}\log\left[1+\frac{\eta +\theta (y_{i}-\eta )}{\beta }\right]\right\}\right],$$

(39) $$P(\beta |x,y)\propto \beta^{-(m+n)+a_{3}-1}\exp\left[-b_{3}\beta \right]\times \exp\left[-(\alpha_{1}+1)\left\{\sum_{i=1}^{J_{1}}\log\left[1+\frac{x_{i}}{\beta }\right]+\sum_{i=J_{1}+1}^{m}\log\left[1+\frac{\eta +\theta (x_{i}-\eta )}{\beta }\right]\right\}\right]\times \exp\left[-(\alpha_{2}+1)\left\{\sum_{i=1}^{J_{2}}\log\left[1+\frac{y_{i}}{\beta }\right]+\sum_{i=J_{1}+1}^{m}\log\left[1+\frac{\eta +\theta (y_{i}-\eta )}{\beta }\right]\right\}\right],$$

(40) $$P(\theta |x,y)\propto \theta^{m+n-(J_{1}+J_{2})-1}\exp\left[-(\alpha_{1}+1)\sum_{i=J_{1}+1}^{m}\log\left[1+\frac{\eta +\theta (x_{i}-\eta )}{\beta }\right]\right]\times \exp\left[-(\alpha_{2}+1)+\sum_{i=J_{1}+1}^{m}\log\left[1+\frac{\eta +\theta (y_{i}-\eta )}{\beta }\right]\right].$$

The joint posterior distribution gives two gamma full conditional distributions are given by (37) and (38) and two general functions of *β* and *θ* (39) and (40), respectively. The posterior distribution (36) can be replaced by the empirical one obtained from data generated from full conditional distribution as the following algorithms.

### 5.1 The empirical posterior under MCMC approach

Several sub-classes of the MCMC approach can be used to simulate the posterior distribution. Gbbis approaches, and general MH, applying Gbbis algorithms are more fitting for the problem at hand; see Metropolis et al. [43] described as follows

**Algorithm 3 (MH under Gibbs algorithms to simulate the posterior distribution)**

1. Begin with indicator κ=1 and entail value $\Theta^{\left(0\right)}=\{{\overset{ˆ}{\alpha }}_{1}$, ${\overset{ˆ}{\alpha }}_{2}$, $\overset{ˆ}{\beta },\overset{ˆ}{\theta }\}$.
2. Generate two values $\alpha_{1}^{\left(\kappa \right)}$, $\alpha_{2}^{\left(\kappa \right)}$ from gamma distribution (37) and (38).
3. Generate two values $\beta^{\left(\kappa \right)}$, $\theta^{\left(\kappa \right)}$ from (39) and (40) using MH algorithms with normal proposal distribution.
4. The indicator κ=κ+1.
5. Replicate steps from 2 to 4 **MC** times and reported the vector $\Theta^{\left(i\right)}=\{\alpha_{1}^{\left(i\right)}$, $\alpha_{2}^{\left(i\right)}$, $\beta^{\left(i\right)},\theta^{\left(i\right)}\}$, i=1,2,…, **MC**.
1. Test the convergence of MCMC to determine ${\mathrm{MC}}^{⁎}$ which need to reach the stationary distribution.

### 5.2 Bayesian point and credible interval of R

Algorithm 4 (point and credible interval of *R*)

1. Compute $R^{\left(i\right)}=\frac{\alpha_{2}^{\left(i\right)}}{\alpha_{1}^{\left(i\right)}+\alpha_{2}^{\left(i\right)}}$, $i={\mathrm{MC}}^{⁎}+$1, ${\mathrm{MC}}^{⁎}+2,\ldots$, **MC**.
2. Put the value of $R^{\left(i\right)}$, $i={\mathrm{MC}}^{⁎}+$1, ${\mathrm{MC}}^{⁎}+2,\ldots$, **MC** in asseding order to be $R_{\left(i\right)}$, i=1,2,…, ${\mathrm{MC}-\mathrm{MC}}^{⁎}$.
3. The Bayes point computation of $\overset{ˆ}{R}$ with respected to mean square error the loss function is displayed by
  (41) $${\overset{ˆ}{R}}_{B}={\text{E}}_{P}(R|x,y)=\frac{1}{{\mathrm{MC}-\mathrm{MC}}^{⁎}}\sum_{i={\mathrm{MC}}^{⁎}+1}^{\mathrm{MC}}R^{\left(i\right)},$$
  and the related variance is given by
  (42) $$\overset{ˆ}{V_{R}}=\frac{1}{{\mathrm{MC}-\mathrm{MC}}^{⁎}}\sum_{i={\mathrm{MC}}^{⁎}+1}^{\mathrm{MC}}{\left(R^{\left(i\right)}-{\overset{ˆ}{R}}_{B}\right)}^{2}.$$
4. The Bayes (1−γ)100% credible intervals given by
  (43) $$\left(R_{\frac{\gamma }{2}({\mathrm{MC}-\mathrm{MC}}^{⁎})},\;R_{\left(1-\frac{\gamma }{2})({\mathrm{MC}-\mathrm{MC}}^{⁎}\right)}\right).$$

## 6 Illustrative examples

Estimation results discussed in this article of *R* are evaluated and compared via a Markov chain Monte Carlo simulation study. Under partial step-stress ALT, the point estimate of the *R* using ML, bootstrap, and Bayes techniques is studied. Also, the approximate confidence interval (ACI), two bootstrap confidence intervals (CIs), and Bayes credible intervals (BCI) of *R*. The simulation case study is conducted for different values of acceleration factor *θ* and different stress change time *η*. In our study, we adopt several values of parameters value and different sample sizes. The point estimates are computed under mean and mean squared error (MSE). However, confidence intervals were tested under mean interval length (MIL) and coverage percentage (CP). For samples, we adopt the size (m,n)={(20, 20), (20, 30), (30, 20), (40, 40), (50, 50)}. For each parameter, the values generate 1000 different samples (Table 1). In the Bayesian approach, 11000 MCMC sample of estimates delete the first 1000 as burn-in and prior information are taken to near the prior mean.

**Table 1:** Different choice of the parameter in simulation study.

case number	*α*_1_	*α*_2_	*β*	*θ*	*η*	*R*
Case I	1.5	0.7	1.0	1.5	1.5	0.318
Case II	1.5	1.2	1.0	2.0	2.0	0.444
Case III	1.5	2.5	1.0	2.0	2.0	0.625
Case V	0.5	2.5	1.0	2.5	1.5	0.833

## 7 Data analysis

The simulated data sets generated from LD(1.5, 3.0) and (3.0, 3.0) to discuss the inference procedures are considered. Under consideration that m=n=25, LD(1.5, 3.0) and (3.0, 3.0) the generated strength data given {0.01425, 0.0315938, 0.103287, 0.325332, 0.369032, 0.524711, 0.593631, 0.652659, 0.722925, 0.753973, 0.971605, 1.47341, 1.55861, 1.87421, 2.09205, 2.23523, 2.31343, 2.6405, 3.08378, 3.19949, 5.17606, 13.0004, 38.4985, 60.9935, 1185.19} and stress data {0.0136923, 0.0329695, 0.14348, 0.176145, 0.238614, 0.363721, 0.499429, 0.517364, 0.55199, 0.672823, 0.708145, 0.709229, 0.833397, 0.974792, 1.41352, 1.44154, 1.57642, 1.84768, 2.23734, 2.88801, 2.92577, 5.66305, 6.45539, 7.44808, 10.9131}, η=3.0. The data for given accelerated factor θ=2.0 and the change stress time *η* are presented in Table 6, Table 7. From the strength data **X** and stress data *Y* the two integer $J_{1}=18$ and $J_{2}=21$. The point estimate of *R* under ML and Bayes method and the related ACI, Boot-p CI, boot-t CI, and BCI are reported in Table 8.

## 8 Conclusions

This paper addresses the issue of ensuring the reliability of a system under stress, considering two variable factors: strength and stress levels during partially step-stress accelerated life testing (ALT). The lifetime distribution for both strength and stress in this scenario follows the Lomax distribution. The main objective is to estimate the reliability factor, denoted as ‘R,’ using various point and interval estimation methods. Extensive simulations were studied to assess the accuracy of these estimations, revealing the following numerical outcomes:

1. The proposed system reliability assessment model performs effectively in all scenarios.
2. Table 2, Table 3, Table 4, Table 5 illustrate that increasing the sample size reduces both mean squared errors (MSEs) and average intervals.

**Table 2:** The mean, MSE, MIL and CP of different estimate of *R* case I.

(*m*, *n*)	Test	*R*_ML_	*R*_Bayes_	Test	ACI	CI_Boot-p_	CI_Boot-t_	BCI
(20,20)	Mean	0.305	0.309	MIL	0.547	0.588	0.525	0.501
	MSE	0.0452	0.0347	CP	(0.89)	(0.90)	(0.91)	(0.90)
(20,30)	Mean	0.309	0.311	MIL	0.521	0.552	0.504	0.489
	MSE	0.041	0.0312	CP	(0.90)	(0.92)	(0.91)	(0.92)
(30,20)	Mean	0.308	0.312	MIL	0.525	0.550	0.511	0.484
	MSE	0.0423	0.0317	CP	(0.91)	(0.91)	(0.93)	(0.94)
(40,40)	Mean	0.312	0.313	MIL	0.511	0.524	0.501	0.455
	MSE	0.0314	0.0243	CP	(0.93)	(0.94)	(0.96)	(0.93)
(50,50)	Mean	0.313	0.314	MIL	0.510	0.511	0.476	0.424
	MSE	0.0252	0.0191	CP	(0.92)	(0.93)	(0.93)	(0.95)
(70,70)	Mean	0.315	0.317	MIL	0.495	0.501	0.451	0.414
	MSE	0.0195	0.0174	CP	(0.93)	(0.96)	(0.92)	(0.93)

**Table 3:** The mean, MSE, MIL and CP of different estimate of *R* case II.

(*m*, *n*)	Test	*R*_ML_	*R*_Bayes_	Test	ACI	CI_Boot-p_	CI_Boot-t_	BCI
(20,20)	Mean	0.421	0.428	MIL	0.785	0.821	0.750	0.711
	MSE	0.0673	0.0558	CP	(0.90)	(0.91)	(0.91)	(0.92)
(20,30)	Mean	0.426	0.431	MIL	0.764	0.803	0.727	0.700
	MSE	0.0623	0.0521	CP	(0.91)	(0.92)	(0.93)	(0.92)
(30,20)	Mean	0.426	0.431	MIL	0.767	0.801	0.722	0.703
	MSE	0.0602	0.0500	CP	(0.90)	(0.92)	(0.90)	(0.94)
(40,40)	Mean	0.429	0.438	MIL	0.741	0.792	0.704	0.682
	MSE	0.0542	0.0462	CP	(0.93)	(0.92)	(0.92)	(0.93)
(50,50)	Mean	0.432	0.440	MIL	0.713	0.769	0.689	0.654
	MSE	0.0501	0.0434	CP	(0.92)	(0.93)	(0.94)	(0.96)
(70,70)	Mean	0.438	0.442	MIL	0.700	0.739	0.652	0.625
	MSE	0.0475	0.0411	CP	(0.93)	(0.93)	(0.92)	(0.93)

**Table 4:** The mean, MSE, MIL and CP of different estimate of *R* case III.

(*m*, *n*)	Test	*R*_ML_	*R*_Bayes_	Test	ACI	CI_Boot-p_	CI_Boot-t_	BCI
(20,20)	Mean	0.604	0.612	MIL	0.725	0.775	0.714	0.685
	MSE	0.0583	0.0511	CP	(0.88)	(0.92)	(0.90)	(0.93)
(20,30)	Mean	0.612	0.628	MIL	0.711	0.766	0.702	0.671
	MSE	0.0555	0.0482	CP	(0.92)	(0.93)	(0.93)	(0.93)
(30,20)	Mean	0.615	0.631	MIL	0.705	0.762	0.705	0.676
	MSE	0.0550	0.0479	CP	(0.92)	(0.92)	(0.93)	(0.95)
(40,40)	Mean	0.619	0.614	MIL	0.689	0.724	0.671	0.645
	MSE	0.0529	0.0451	CP	(0.94)	(0.92)	(0.94)	(0.96)
(50,50)	Mean	0.620	0.619	MIL	0.665	0.700	0.638	0.614
	MSE	0.0504	0.0424	CP	(0.92)	(0.96)	(0.93)	(0.94)
(70,70)	Mean	0.622	0.625	MIL	0.665	0.681	0.614	0.600
	MSE	0.0481	0.0403	CP	(0.93)	(0.95)	(0.93)	(0.94)

**Table 5:** The mean, MSE, MIL and CP of different estimate of *R* case V.

(*m*, *n*)	Test	*R*_ML_	*R*_Bayes_	Test	ACI	CI_Boot-p_	CI_Boot-t_	BCI
(20,20)	Mean	0.817	0.819	MIL	0.873	0.899	0.855	0.832
	MSE	0.0621	0.0575	CP	(0.90)	(0.91)	(0.90)	(0.92)
(20,30)	Mean	0.818	0.819	MIL	0.851	0.868	0.828	0.814
	MSE	0.0603	0.0559	CP	(0.92)	(0.91)	(0.93)	(0.94)
(30,20)	Mean	0.817	0.820	MIL	0.855	0.868	0.822	0.810
	MSE	0.0607	0.0556	CP	(0.93)	(0.94)	(0.93)	(0.93)
(40,40)	Mean	0.821	0.822	MIL	0.827	0.851	0.803	0.785
	MSE	0.0589	0.0524	CP	(0.92)	(0.92)	(0.93)	(0.92)
(50,50)	Mean	0.819	0.825	MIL	0.800	0.817	0.781	0.749
	MSE	0.0561	0.0503	CP	(0.93)	(0.92)	(0.92)	(0.96)
(70,70)	Mean	0.826	0.830	MIL	0.786	0.805	0.766	0.724
	MSE	0.0525	0.0491	CP	(0.91)	(0.92)	(0.93)	(0.95)

**Table 6:** Strength data under partially step-stress ALT (**X**).

0.01425	0.0315938	0.103287	0.325332	0.369032	0.524711	0.593631	0.652659	0.722925
0.753973	0.971605	1.47341	1.55861	1.87421	2.09205	2.23523	2.31343	2.6405
3.04189	3.09974	4.08803	8.00021	20.7493	31.9967	594.096

**Table 7:** Stress data under partially step-stress ALT (**Y**).

0.0136923	0.0329695	0.14348	0.176145	0.238614	0.363721	0.499429	0.517364	0.55199
0.672823	0.708145	0.709229	0.833397	0.974792	1.41352	1.44154	1.57642	1.84768
2.23734	2.88801	2.92577	4.33153	4.7277	5.22404	6.95654

**Table 8:** The point and interval estimate of (**R**).

${\overset{ˆ}{R}}_{\text{Exact}}$	${\overset{ˆ}{R}}_{\text{ML}}$	${\overset{ˆ}{R}}_{\text{Bayes}}$	ACI	Boot-p CI	Boot-p CI	BCI
0.667	0.604	0.623	(0.472, 0.737)	(0.415, 0.812)	(0.499, 0.715)	(0.521, 0.707)
3. Bootstrap-t and Bayesian techniques outperform MLE and bootstrap-p methods.
4. Confidence intervals (CPs) closely approximate the proposed one, especially with a large sample size.
5. The suggested inference methods for ‘R’ consistently yield reliable outcomes.
6. The estimation results gets better for large value of *η*.

## CRediT authorship contribution statement

**Hassan M. Aljohani:** Formal analysis, Data curation, Conceptualization.

## Declaration of Competing Interest

There is no conflict of interest regarding publishing this paper.

## Data Availability

All data are available in the paper' with its related references.
